# Stakeholder engagement as a valuable tool to improve the relevance of research regarding framework development

**DOI:** 10.1038/s44433-026-00006-9

**Published:** 2026-06-03

**Authors:** Rosemary A. Reyneke, Imogen F. Richens, Heather Buchanan, E. Bethan Davies, Marnie L. Brennan

**Affiliations:** 1 Centre for Evidence-based Veterinary Medicine, School of Veterinary Medicine and Science, The University of Nottingham, Nottingham, UK; 2 VetPartners, Spitfire House, Aviator Court, York, UK; 3 Lifespan & Population Health, School of Medicine, The University of Nottingham, Nottingham, UK; 4 NIHR MindTech Medtech Co-operative, Institute of Mental Health, University of Nottingham, Nottingham, UK

**Keywords:** Health care, Medical research

## Abstract

This paper presents a stakeholder engagement process conducted as part of a larger research project to develop a new framework to support the adoption of preventive medicine practices on ruminant farms. Stakeholder engagement enhances research relevance by aligning it with the needs of end users. While common in human healthcare, it is less prevalent in veterinary medicine. Stakeholders engaged in this work included future end users—veterinarians and livestock farmers. A combination of approaches was utilised, including in-person and online group discussions and individual conversations. Stakeholder contributions significantly shaped the research—both the content and structure of the framework, as well as the methods used to develop it. Their involvement improved the framework’s practical relevance and usability, increasing the likelihood of future adoption. The findings demonstrate that stakeholder engagement is not only valuable but also feasible within veterinary research settings.

## Introduction

To maintain and grow research in veterinary medicine, it is critical to ensure that the research work conducted is relevant and transparent. Stakeholder input provides a valuable tool in ensuring research is suitable and focuses on what is of value to the stakeholder^1,2^. Engaging stakeholders in research design helps prioritise outcomes that reflect real-world demands^1,2^. Furthermore, stakeholder engagement aids the design and conduct of trials that reflect real-world conditions, which in themselves enhance translational impact^1^. Stakeholder engagement can, and should, form a central role in the research process, improving the value of the work being done.

Stakeholders have been defined as:

“*individuals, organizations or communities that have a direct interest in the process and outcomes of a project, research or policy endeavour*”^3^ (p. 5).

With engagement being:

*“an iterative process of actively soliciting the knowledge, experience, judgment and values of individuals selected to represent a broad range of direct interest in a particular issue, for the dual purposes of: creating a shared understanding; making relevant, transparent and effective decisions”*^3^ (p. 5).

Stakeholder engagement has long been acknowledged as a critical component in human healthcare research, with growing international consensus on the necessity of involving stakeholders throughout the research process^4^. As an example, in the United Kingdom, national governance frameworks mandate that researchers work *with* members of the public, rather than conducting research *on* them^5^. This focus is further reinforced by major UK health research funders, who have formally integrated public and patient involvement (PPI) into their funding criteria and evaluation processes^6^. The incorporation of stakeholder engagement has reshaped research priorities, enhanced participant recruitment methods, improved consent materials, and ensured outcomes reflect patient needs^5,7–9^.

However, whereas stakeholder engagement has become commonplace in human healthcare^4^, the value of stakeholder engagement appears to be earlier in the journey towards widespread acceptance in veterinary research. That is not to say it is not being done at all—there are a growing number of valuable examples where perspectives of stakeholders have been gathered, including through workshops^10^, interviews^11^ or surveys^12^, as well as through innovative approaches such as Living Labs^13^. However, it is important to understand, and to highlight, the difference between a stakeholder engagement process as described here, and qualitative research approaches illustrated in the examples above. The former seeks input to shape the research being done, whilst the latter engages with people to specifically answer a research question. The key differences are summarised in Table 1.

**Table 1:** Key differences between stakeholder engagement and qualitative research, adapted from ref. ^58^

	Stakeholder engagement	Qualitative
Research question	Aims to help select and refine a research question	Aims to answer a predefined research question
Ethical approval	Needs to reflect ethical practices, but does not need ethical approval	Requires ethical approval from the ethics board
People’s input	Seeks input to inform and influence decisions about how research is designed and undertaken	Seeks people’s input to help build an argument and answer a research question
Power	Views from stakeholders and researchers are combined to make joint decisions	Only researchers have the power to make decisions about the project’s design
Purpose	Increases relevance through active involvement in decisions about research priorities, design and conduct	Advances understanding and generates new knowledge using standardised methods for skilled data collection from research participants

The research questions asked in qualitative research can, and indeed should, directly inform the design of research^10,13^. However, they require formal methods and analysis that can be time-consuming and resource-hungry. Furthermore, although qualitative research gives voice to individuals, it does not allow them to directly influence the research process or subsequent steps. Instead, stakeholder engagement provides an approach that supports gaining valuable input from stakeholders in a pragmatic manner and furthermore, aids research relevance by including stakeholders actively in the decision-making process.

Further exploration into both the advantages of stakeholder engagement and the methodologies by which it can be effectively implemented in veterinary contexts would be beneficial to support more relevant and impactful research practices. This paper outlines a process of stakeholder engagement conducted as part of a research project aimed at developing a novel framework to enhance the adoption of preventive medicine practices on ruminant farms. Frameworks, derived from fields such as human behaviour change and implementation science, have been widely utilised in human healthcare and provide a valuable tool to guide the implementation of evidence-based practices^14–16^. However, their application in veterinary or agricultural contexts remains limited^17^.

This study aims to utilise stakeholder engagement early on in the research process to improve the relevance and practical applicability of the framework being developed, and to support its eventual integration into real-world practices.

## Methods

### Context

**Fig. 1:**
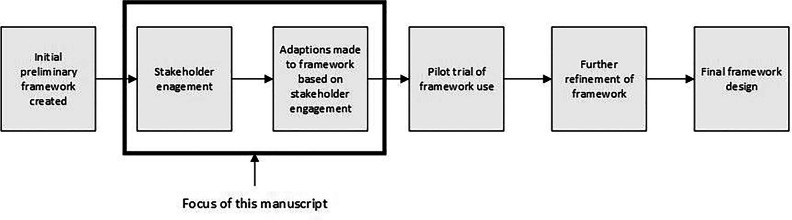
Flowchart showing the plan for the larger research project, highlighting the section of the research described in this manuscript.

Before initiating stakeholder engagement, the research team had developed a preliminary framework by synthesising existing frameworks. Stakeholder engagement was then undertaken to identify potential beneficial modifications that could be made to the preliminary framework, as well as to guide the design of the next stage in framework development, a pilot trial of its use (Fig. 1).

### Participants

Stakeholders included intended end-users for the tool being developed—practising ruminant veterinarians (vets) and ruminant farmers. Furthermore, individuals with expertise in the topic area—those with experience in encouraging adoption of practices or introducing change, either in a veterinary or non-veterinary context were also consulted. This latter group is hereafter referred to as “change agents”^18^.

### Recruitment

Vets were recruited through several approaches, including existing contacts, emails to local veterinary practices, and attendees at a conference workshop. Farmers were recruited through existing contacts and attendees at a livestock market. Change agents were initially recruited through existing contacts, then following an iterative snowball recruitment process as consultees suggested further individuals that would be suitable for participation.

Stakeholder engagement to inform a research process is not considered data collection^19^. Therefore, unlike some forms of qualitative research^20^, ensuring appropriate sample size and seeking data saturation are not formal components—the purpose is to enhance study design rather than to generate saturable data. However, contemporary qualitative evidence suggests that four focus groups or nine interviews are typically suitable for theme saturation to occur^21^, and this was seen as a pragmatic benchmark for ensuring diverse viewpoints without unnecessary burden. Ultimately, the process was guided by iteratively exploring a range of perspectives and experience, with the decision to conclude the consultation process being guided predominantly by the researcher’s judgement that a broad range of perspectives had been gathered, and further discussions would likely yield minimal additional insights^22^.

Potential consultees were given a brief overview of the project area and the purpose of the consultations to gain their agreement to be part of the process.

### Eligibility criteria

There were no specific eligibility criteria, except that participants fell into one of the classifications as described above. All individuals who expressed interest in the consultations were included in the process.

### Format of consultations

Consultations were conducted between October 2023 and May 2024. No honorarium was provided for participation, though lunch or snacks were offered during in-person meetings held at veterinary practices. The consultations took various formats influenced by the relevant topics for exploration at the time of the consultation and were held in different locations based on pragmatic logistics and participant preferences. The approach used varied between semi-structured and unstructured, which was dictated by both pragmatic factors, such as time available, alongside the type of consultation. Consultations with more than two individuals followed a more structured approach to ensure that discussions remained focused on issues of relevance^23^, whereas individual and pair conversations were unstructured, allowing flexibility to move away from researcher-defined assumptions^24^. Full details of the format of the consultations are shown in Table 2.

**Table 2:** Format of stakeholder engagement undertaken to aid development of a novel framework to support uptake of preventive medicine practices on ruminant farms

Consultation format	Date(s)	Participants	Number of participants	Location	Content
In person workshop at a veterinary conference (*n* = 1)	October 2023	Attendees at the conference	19	Annual veterinary conference	Researcher (RR) gave an introduction to the concept of using frameworks to support uptake of evidence-based practices, followed by small group discussions on experiences of barriers and facilitators.
Online focus group (*n* = 1)	February 2024	Participants from the conference workshop—veterinarians	3	Online (Microsoft Teams)	A semi-structured interview guide was followed, including asking what factors participants felt influenced the use of a framework-based approach to encourage uptake of evidence-based practices on farms.
In-person group discussions (*n* = 4)	February to April 2024	Veterinarians	19	Veterinary practices	A semi-structured interview guide was used. The discussions were divided into two parts: the first explored perceived issues hindering the uptake of preventive medicine practices on farms, and the second (following a more informal format) introduced the concept of the framework and sought feedback on its potential utility.
In-person group discussion (*n* = 1)	March 2024	Farmers	8	Social setting, as part of a monthly group meeting	The focus was on identifying barriers to the uptake of preventive medicine practices and understanding current veterinary-farmer interactions regarding preventive health. While the concept of the research project was introduced, feedback on the framework was not solicited due to time limitations.
Individual conversations (*n* = 2)	January and February 2024	Veterinarians	2	Online (Microsoft Teams)	Followed an unstructured format, with the aim to gain a broader understanding of existing practices and challenges.
Individual/pair conversations (*n* = 8)	December 2023 to May 2024	Change agents	10	Online (Microsoft Teams)	Followed an unstructured format, included discussions around individuals’ experiences with enacting change, factors they viewed as valuable to the process, and, in some cases, feedback on the proposed framework-based approach.
Individual/pair conversations (*n* = 3)	April 2024	Farmers	4	In-person—livestock market	These brief unstructured discussions focused on the challenges of preventive medicine uptake.

### Interpretation of consultations

The approach to interpreting the feedback from consultations was adapted from established methods used in patient and public involvement (PPI), a practice widely used in human healthcare^25^ and was guided by the Oxford PPI recording tool (available here: https://www.phc.ox.ac.uk/files/ppi/ppi-recording-tool-1_august23.docx).

For all consultation formats, the lead researcher (RR) typed notes on points raised and then categorised each point under a relevant broad topic based on the researcher’s judgement. The note of what was said in each consultation and the broad topic it came under was then transferred into a Microsoft Excel document, and related points from different consultations were grouped together within the same topic area. Subsequently, each identified topic, or individual point, was assessed in relation to the pre-existing framework and research plan. The researcher then decided whether any alterations to the framework or research plan were warranted based on the feedback received. Any modifications made were documented on the Excel spreadsheet, including the rationale behind the decision to incorporate or disregard specific feedback.

In recording the interpretations of the consultations, three key points were systematically documented:

1. What did contributors say or suggest?
2. As a result, what feedback was incorporated or what was changed in the study?
3. Why were their suggestions or feedback incorporated, or why not?

Rarely, there were cases where what was said by different individuals was contradictory. Given the aim of the engagement was to appreciate a range of opinions, if a point was deemed important by one individual it was included, regardless of whether another individual expressed that it was less, or not, important. It is a key aspect of stakeholder engagement that all input should be considered, even if it comes from a single source, especially when the aim is to understand a range of perspectives^22^.

The study was reported on using the Guidance for Reporting Involvement of Patients and the Public (GRIPP 2—long form) to enhance the quality and transparency of stakeholder engagement reporting^26^ (see Supplementary file).

### Researcher positionality statement

RR is a qualified veterinarian with 15 years of experience working with ruminant farmers. Starting her PhD in 2021 was her first experience of focused research work, with this article describing her first formal experience of stakeholder engagement to inform research. MB and IR are qualified veterinarians and researchers, with experience in a variety of qualitative research methods, including with ruminant farmers and veterinarians. HB and EBD are psychologists specialising in health psychology and mental health research. They have extensive experience working in research with expertise including behaviour change and qualitative approaches to data collection. EBD has 8 years of experience in PPI-related activities.

### Ethics and consent to participate

The conduct of stakeholder engagement to inform a research process (rather than to answer a specific research question) does not require ethical approval as it is considered a consultative process rather than generalisable research involving human participants (PPI Ignite network statement—https://ppinetwork.ie/resource/ppi-ignite-network-statement-on-research-ethics-committee-approval/).

However, for this project—participants in the conference workshop, online and vets’ in person focus groups were informed about the purpose, procedures, potential risks, and benefits of the research before providing written consent, with ethical approval being granted by the Committee for Animal Research & Ethics, School of Veterinary Medicine and Science, University of Nottingham, approval number 3961 231003. All other stakeholders gave verbal consent to be consulted, but are not considered research participants or data providers, in line with the PPI Ignite Network Statement on research ethics committee approval. All research activities involving human participants were conducted in accordance with the ethical standards of the Committee for Animal Research & Ethics, School of Veterinary Medicine and Science, University of Nottingham, and NIHR Standards for Public Involvement^27^, with all human participants providing consent to participate. Clinical trial number: Not applicable.

## Results

### Context

Consultations with vets and change agents encompassed both the identification of barriers to the adoption of preventive medicine practices and the elicitation of feedback on a framework-based approach. In contrast, due to pragmatic considerations—primarily time constraints—consultations with farmers were focused predominantly on identifying barriers to the uptake of preventive medicine practices, without direct exploration of the framework itself. Consequently, as this manuscript concentrates on informing the development of the framework-based approach, farmer-derived feedback is comparatively limited relative to that obtained from other participant groups.

### Participants

Full details of participants and format of engagement can be found in Table 2.

### Influence on the framework being developed

The stakeholder feedback significantly influenced the specific design and components of the framework under development. Insights from the consultations reinforced the value of existing elements of the initial draft framework, while also prompting changes. These changes included modifications to the framework’s composition, such as:

- Adjustments or additions to framework components.
- Terminology used in the framework.
- Format of the framework—to align with how it would be used and the natural progression of topics.

Examples of what was said, the changes made, and the rationale behind them can be seen in Table 3.

**Table 3:** Influence of feedback from participants as part of stakeholder engagement on the specific design and components of the framework under development

Area of framework altered	What did contributors say or suggest?		As a result, what feedback was incorporated or changed in the study?	Why were their suggestions or feedback (not) incorporated?
	Who	What was said		
Ensuring framework components are relevant	Vet	Best to specify separate short- and long-term goals	Redefined a new construct “define goals” with detail added to the specific need to define short-, medium- and long-term goals	Was mentioned by several stakeholders and provided valuable clarity.
	Change agent	Good concept to have short (1 year), medium (2–3 years), and long-term (5 years plus) goals		
	Vet	Narrowing down the priority problem is very important—often lots of different issues on one farm, ideally have this clearer or as a separate step?	Added a new construct “prioritise”, with details around this being a co-productive decision	Was widely recognised to be an important step to take to achieve change.
	Change agent	May be valuable to explore what drives priority for individuals		
	Farmer	Felt vets often focused on what was an assumed priority, without consulting the farmer		
Ensuring framework language is appropriate	Change agent	Questioned terminology of ‘Plan’—that this phase was more about goals/objectives	Redefined what the ‘plan’ construct meant, and added new constructs ‘set objective’ and ‘define goals’	It was important that the terminology is clear, so that is easy to understand.
Ensuring framework is easy to use	Vet	Framework represents a linear process, which may not be what actually happens if things don’t go to plan or they (the farmer) are not as on board as you think they are. Need capacity for backwards steps	The final framework design was adjusted to reflect a non-linear flow	This suggestion reflected real situations, was reinforced by several stakeholders, and was pragmatic to enact.
	Change agent	Framework felt to be too linear, several places where non-linear steps would be expected or needed		
	Vet	Feedback on time needed varied, but the general feeling was that two visits better—the second visit from ‘assess’—this would take most time	Initial first planning phase split into two broad separate components	This suggestion reflected real situations, was reinforced by other stakeholders, and was pragmatic to enact.
	Vet	Familiar farm—may be able to do in one visit, less familiar farm—one visit for explore, focus, plan, second visit for the second half		

### Influence on how the research could be conducted

The consultations provided feedback not just on the design of what was being researched (the framework itself), but also on how it would likely be used, informing the next stage of framework development, a pilot trial of its use. This included:

- What would be needed before the framework could be used—training in its use.
- How the framework might be used—which varied from using it as a broad guide, to having it visible to the farmer, and going through it step by step.
- How/where to follow the framework—including location of likely discussions using the framework, as well as what time may be needed and what time may be available.
- Who needs to be involved when the framework is used.
- What supporting resources may be needed alongside the framework itself.

Examples of what was said, the changes made, and the rationale behind them can be seen in Table 4.

**Table 4:** Influence of feedback from participants as part of stakeholder engagement on how a pilot trial exploring the use of the novel framework could be conducted

Area of framework altered	What did contributors say or suggest?		As a result, what feedback did you incorporate or change in the study?	Why did or didn’t you incorporate their suggestions or feedback?
	Who	What was said		
What needs to happen before the framework is used	Vet	Vets currently lack the required communication skills	Vets will receive some training in how to use the framework before the pilot trial, but researchers need to be aware that this level of training may be insufficient.	The ability to provide the comprehensive training that may be required was beyond the pragmatic capabilities of this project.
	Vet	Would need to practice it quite a lot to be able to deliver it in a meeting (especially with less engaged farmers).		
How the framework may be used	Vet	“if you had the framework in your head, and you were like, right I’m going to talk, initially, the first couple of minutes I’m going to talk about the planning, and then I’m going to move round it. I could see it working”	For the pilot trial of framework use, ensured there were no specific guidelines about how to follow the framework, just that the framework had to be followed. This should allow for different preferences on approaches.	To effectively assess framework use, it needs to be done in a way that reflects how that framework will be used outside of a controlled research environment.
	Vet	May be good to have a questionnaire alongside it, so you know you’re asking all the right questions		
How and/or where to utilise the framework	Farmer	Benefit of chatting during TB test is you’re with the stock, and each individual one, whereas farmers felt when they were in sit down meetings, they tended to forget things.	Recommendation that discussion, where possible, be conducted on farm, ideally while walking around, rather than in a meeting room.	Effective engagement in the framework is key to realise potential efficacy of the framework, therefore important that approach adopted maximises likely engagement.
	Change agent	Getting out and looking at what you’re asking questions about (i.e. the farm, the animals) is of great value		
	Vet	Ideally not too long a conversation—something that may take 1 h most days but can be done in 20 min if needs be.	Recommendation and intent that each section of the framework that is designed to be conducted at once should be able to be covered in a discussion lasting no longer than 1–2 h.	The necessary process of following the framework can be time consuming, but this is recognised as a serious barrier to use, therefore to support actual uptake, practical time constraints needed to be taken into consideration.
Who needs to be involved	Vet	“Another thing at which people can improve at … is to talk to the people lower down, because they’ll probably be more honest with you, to find your problems and things”	Incorporate into the framework the concept of identifying the right people to be involved in the discussion, getting them involved and then including them in discussions and decisions about what changes will be made.	It was widely felt that not addressing this issue was a common source of failure to change, and was something that should be addressed.
	Vet	“You might just be talking to the herd manager, and actually it’s going to be someone else that at 3 in the morning is going to be calving the cows and dipping the navels in iodine. “		

### What was said by stakeholders that did not result in change, and why

There were points raised during the consultations that did not result in changes to the research plan. The reasons for this fell into two broad categories:

- What was said was already in the research plan.
- What was said was considered valuable, but not able to be incorporated into the framework or the approach at this stage, chiefly due to practical constraints.

Examples of what was said but that did not lead to changes, and the rationale behind this can be seen in Table 5.

**Table 5:** Feedback from participants as part of stakeholder engagement that did not result in a change to framework development or conduct of further research

Reason why changes were not made as a result of what was said	What did contributors say or suggest?		As a result, what feedback did you incorporate or change in the study?	Why did or didn’t you incorporate their suggestions or feedback?
	Who	What was said		
Already part of the research plan	Vet	“We all go round believing that NSAIDs cost and farmers won’t use them to treat cows with lameness, and then the farmers all basically have told the survey that they don’t see cost as a barrier to using NSAIDs in the treatment of lameness. So vets and farmers are saying the complete opposite thing.”	No change made	There is already a specific step in the framework around exploring barriers and facilitators.
	Farmer	Vets sometimes may not give clear information regarding, for example, vaccine timing, which can impact efficacy of vaccine.	No change made	It is already within the framework the need for detailed understanding and checking understanding between parties.
	Vet	Things can go wrong if farmers don’t fully understand what they have been told		
Not feasible to incorporate at this stage	Vet	“As vets we can be really really technical when we want to be, and trying to distil out the key information and also take it into, well not necessarily lay terms, but certainly less technical terms, is often a challenge”	No change made	Beyond the scope of the framework approach to be able to control this, but is incorporated to some extent into training given to vets before using framework.
	Vet	Vets asking direct specific questions, rather than broad open questions—and so missing important bits of information and not encouraging sharing.		

### Wider impact of consultations

The consultations had a broader impact beyond the areas already highlighted:

- Engaging with stakeholders early on and involving them in the research process facilitated the recruitment for the next phase of the study—the pilot trial of the framework’s use. Notably, two of the veterinarians who participated in the consultations later became participants in the ongoing small-scale pilot trial.
- Discussions with a diverse group of stakeholders revealed a wide array of potential applications for the framework. These included its integration with existing on-farm discussions, such as England’s DEFRA funded Animal Health and Welfare Pathway (https://www.gov.uk/government/publications/animal-health-and-welfare-pathway/animal-health-and-welfare-pathway) or the TB advisory service (TBAS, https://www.tbas.org.uk/). This broader perspective has expanded the potential reach and utility of the framework, suggesting that it could complement and enhance current practices in various contexts out with the original intended focus.

## Discussion

In this work, stakeholder engagement played a critical role in shaping the research process. This collaborative approach has increased the likelihood of the framework’s acceptability, successful adoption, and broader impact. The stakeholder engagement:

- Shaped the development of a valuable and practical tool (framework) in a way that is anticipated to increase both the adoption rate and the effectiveness of the framework-based approach. Improvements were made in terms of relevance, language clarity, alignment with user needs, and overall usability, all of which have been shown to be pivotal in effective framework application^28,29^.
- Contributed to the design of a relevant pilot trial. The feedback gathered provided valuable insights into how the framework may be implemented in real-world scenarios, ensuring that a pilot trial of its use would be conducted in a way that would closely reflect how it would ultimately be used.

Stakeholder engagement has been widely reported to impact multiple aspects of a project, being considered by both researchers and stakeholders to be worthwhile^30^. Studies have reported that stakeholder input refines research questions, protocols, and interventions^31–33^. Stakeholder contributions improve study design, recruitment strategies, and measurement tools so that research better reflects participant needs, leading to improved study feasibility, acceptability, and relevance^34,35^. There are also long-term benefits of stakeholder engagement, which are beginning to be realised by this project, such as building trust and strengthening long‐term research partnerships^36,37^. These are key benefits that improve the research environment, enhance sustained collaborative relationships and support ethical practice through greater community autonomy and fair benefit-sharing^38^. Key enablers to support effective long-term research partnerships include trust, communication skills, remuneration, and dedicated time investment^39^.

A core strength of this work was the breadth of stakeholders engaged with, alongside the collaborative nature of this engagement, including active discussion around influencing the research process. The value of these aspects has been well documented in the literature, including engaging with different end-users^40^ and topic specialists^41–43^, and utilising a co-productive approach^13,44–46^.

However, the work did have some limitations. In contrast to human healthcare, where patient and public involvement (PPI) is supported by established frameworks and widely recognised practices^25^, veterinary medicine lacks a similarly well-defined pathway. As a result, researchers engaged with stakeholders without the benefit of a standardised template or familiar process to guide either party. This absence of precedent may have influenced both the nature and extent of engagement. For example, recruitment of farmers proved challenging, likely partially because the stakeholder consultations were occurring predominantly during spring when livestock farmers are particularly busy, potentially limiting their availability for research engagement^47,48^. Furthermore, engagement with vets was supported through aligning with the existing format of regular veterinary practice meetings, whereas small group discussions with a number of farmers are not as commonplace and therefore more creative approaches to engagement were undertaken, such as consultation with attendees at a livestock market.

The research project was already underway when the concept of stakeholder engagement was considered. As such, what was presented at consultations and discussed with stakeholders was only a portion of the research project, whereas success from stakeholder engagement is most evident when engagement occurs early, and is sustained throughout the process^36,37^. Having said this, although stakeholders were not involved from the start in this work, the value of consulting with them was recognised prior to implementing the pilot trial, which was a key stage in the research plan.

This study focused particularly on engaging with future end-users; however, stakeholders are not just limited to end-users and topic specialists, and it is most beneficial to engage with a wide pool of stakeholders^49^. Those working in human healthcare propose a concept of the “7Ps of stakeholders”—namely, providers, policy makers, payers, purchasers, product makers, principal investigators (researchers), and patients/public^50^, which may form the basis of a useful framework for identifying relevant stakeholders to veterinary research. In future, engagement could be expanded to include other on farm workers^51^ and advisors^52^ as well as wider industry bodies and training bodies (for example, universities and colleges offering training for vets and farmers) to further ensure that what is being developed is appropriate, usable and aligns with the interests and needs of the industry, as well as of the direct end-users.

Consultation approaches used were varied and flexible, which aided recruitment and supported open engagement. Stakeholder engagement can be conducted through a wide range of approaches, including focus groups^53^, stakeholders as research partners^31,54^, workshops^55^ and advisory or steering committee groups^31,32,56^, with many studies using more than one method of engagement^30,32^. As done here, it should be flexible to suit the needs of the stakeholder^57^, as well as the researchers.

Collaborative work between researchers and stakeholders can involve various levels of interaction and exchange, as detailed in Table 6. In this work, although multiple meaningful changes were made directly as a result of what was said, stakeholders were not consulted directly about what changes were being made, and therefore, there was scope for a greater degree of engagement^58,59^.

**Table 6:** A ladder of stakeholder management and engagement. Adapted from ref. ^59^.

	Degree of engagement	Intention of engagement	Style of dialogue and associated examples
↑ Increasing engagement	Stakeholder control	Majority representation of stakeholders in decision-making process.	Multi-way dialogue, e.g., community projects.
	Partnership	Joint decision-making power over specific projects.	Multi-way dialogue, e.g., joint ventures.
	Collaboration	Some decision-making power afforded to stakeholders over specific projects.	Multi-way dialogue, e.g., strategic alliances.
	Consultation	Organisation has the right to decide. Stakeholders can advise.	Two-way dialogue, e.g., questionnaires, interviews, focus groups, task forces, advisory panels.
	Explaining	Educate stakeholders.	Two-way dialogue, e.g., workshops.
	Informing	Educate stakeholders.	One way dialogue, e.g., verified corporate reports.

In conclusion, stakeholder engagement has been widely shown to be beneficial to the research process and has become embedded in research practices in fields such as human healthcare. Through a process of stakeholder engagement in our current research, we were able to constructively modify both what was being researched as well as how it was being researched to improve the relevance of the work being done, and increase the likelihood of use, and therefore impact, in the real world.

Through a discussion of the stakeholder engagement undertaken, this paper emphasises the critical role stakeholder engagement can play in creating a functional and relevant tool. By describing the process followed and detailing the key lessons learnt, we highlight the value as well as the details of the practical and pragmatic application of this approach. We propose that stakeholder engagement can and should be an integral component of all veterinary research involving intervention or tool development, using approaches such as the Stakeholder Engagement Reporting Questionnaire^60^ or GRIPP2^26^ to guide reporting of this work.

## Supplementary information

Supplementary information

## Data Availability

The data that support the findings of this study are derived from discussions involving human participants. Due to them containing information that could compromise research participant privacy or consent, the datasets are available only on request. Requests for access to researcher notes and, where relevant, anonymized excerpts will be considered on a case-by-case basis. Researchers wishing to access the data must: Submit a formal request outlining the intended use; Agree to use the data solely for scholarly research purposes; Refrain from any attempts to re-identify participants; Comply with all applicable data protection regulations (e.g., GDPR); Sign a Data Use Agreement provided by the data custodian. Requests can be directed through the University of Nottingham data repository—DOI: 10.17639/nott.7614. Requests for access will be reviewed and responded to within 15 business days. Data will be shared subject to ethical approval and a signed Data Use Agreement.
