# Supplementary material for: Stakeholder engagement as a valuable tool to improve the relevance of research regarding framework development
Source: npj Vet Sci. 2026 Jun 3;1(1):10. doi: 10.1038/s44433-026-00006-9 (PMC13233298; doi:10.1038/s44433-026-00006-9)
Supplement: Supplementary file 1 — Supplementary information [file 44433_2026_6_MOESM1_ESM.pdf]

GRIPP2 long reporting form

Section and topic	Item	Reported
Section 1: Abstract of paper		
1a: Aim	Report the aim of the study	Stakeholder engagement process conducted as part of a research project to develop a new framework
1b: Methods	Describe the methods used by which patients and the public were involved	A combination of approaches was utilised, including in person and online group discussions and individual conversations
1c: Results	Report the impacts and outcomes of PPI in the study	Stakeholder contributions significantly shaped both the content and structure of the framework, as well as the methods used to develop it.
1d: Conclusions	Summarise the main conclusions of the study	Their involvement improved the framework's practical relevance and usability, increasing the likelihood of future adoption. The findings demonstrate that stakeholder engagement is not only valuable but also feasible within veterinary research settings.

GRIPP2 long reporting form

**Section and topic**	**Item**	**Reported**
1e: Keywords	Include PPI, "patient and public involvement," or alternative terms as keywords	Stakeholder engagement, co-production
Section 2: Background to paper		
2a: Definition	Report the definition of PPI used in the study and how it links to comparable studies	Definition of stakeholder engagement reported.
2b: Theoretical underpinnings	Report the theoretical rationale and any theoretical influences relating to PPI in the study	None relevant
2c: Concepts and theory development	Report any conceptual or theoretical models, or influences, used in the study	None used
Section 3: Aims of paper		

GRIPP2 long reporting form

Section and topic	Item	Reported
3: Aim	Report the aim of the study	This study aims to utilise stakeholder engagement to aid development of a novel framework to ensure its relevance, practical applicability, and eventual integration into real-world practices.
Section 4: Methods of paper		
4a: Design	Provide a clear description of methods by which patients and the public were involved	Detailed in Table 1
4b: People involved	Provide a description of patients, carers, and the public involved with the PPI activity in the study	Detailed in Table 1
4c: Stages of involvement	Report on how PPI is used at different stages of the study	Reported in 'Context'

GRIPP2 long reporting form

Section and topic	Item	Reported
4d: Level or nature of involvement	Report the level or nature of PPI used at various stages of the study	'Context', Table 1 and 'Interpretation of consultations'
Section 5: Capture or measurement of PPI impact		
5a: Qualitative evidence of impact	If applicable, report the methods used to qualitatively explore the impact of PPI in the study	N/A
5b: Quantitative evidence of impact	If applicable, report the methods used to quantitatively measure or assess the impact of PPI	N/A
5c: Robustness of measure	If applicable, report the rigour of the method used to capture or measure the impact of PPI	N/A
Section 6: Economic assessment		

GRIPP2 long reporting form

Section and topic	Item	Reported
6: Economic assessment	If applicable, report the method used for an economic assessment of PPI	N/A
Section 7: Study results		
7a: Outcomes of PPI	Report the results of PPI in the study, including both positive and negative outcomes	Results section
7b: Impacts of PPI	Report the positive and negative impacts that PPI has had on the research, the individuals involved (including patients and researchers), and wider impacts	Results section
7c: Context of PPI	Report the influence of any contextual factors that enabled or hindered the process or impact of PPI	Results section – 'Context'

GRIPP2 long reporting form

Section and topic	Item	Reported
7d: Process of PPI	Report the influence of any process factors, that enabled or hindered the impact of PPI	Table 1, further explored in the discussion.
7ei: Theory development	Report any conceptual or theoretical development in PPI that have emerged	N/A
7eii: Theory development	Report evaluation of theoretical models, if any	N/A
7f: Measurement	If applicable, report all aspects of instrument development and testing (eg, validity, reliability, feasibility, acceptability, responsiveness, interpretability, appropriateness, precision)	N/A
7 g: Economic assessment	Report any information on the costs or benefit of PPI	Benefits detailed.

GRIPP2 long reporting form

**Section and topic**	**Item**	**Reported**
Section 8: Discussion and conclusions		
8a: Outcomes	Comment on how PPI influenced the study overall. Describe positive and negative effects	Discussion and Results
8b: Impacts	Comment on the different impacts of PPI identified in this study and how they contribute to new knowledge	First 4 paragraphs of discussion section.
8c: Definition	Comment on the definition of PPI used (reported in the Background section) and whether or not you would suggest any changes	Discussion – more detail given around definition of stakeholder, and differing levels of engagement.
8d: Theoretical underpinnings	Comment on any way your study adds to the theoretical development of PPI	N/A
8e: Context	Comment on how context factors influenced PPI in the study	Discussion.

GRIPP2 long reporting form

Section and topic	Item	Reported
8f: Process	Comment on how process factors influenced PPI in the study	Discussion – strengths and limitations around type of stakeholder engaged and stage of research plan where engagement occurred.
8 g: Measurement and capture of PPI impact	If applicable, comment on how well PPI impact was evaluated or measured in the study	N/A
8 h: Economic assessment	If applicable, discuss any aspects of the economic cost or benefit of PPI, particularly any suggestions for future economic modelling.	N/A
8i: Reflections/critical perspective	Comment critically on the study, reflecting on the things that went well and those that did not, so that others can learn from this study	Discussion section
